# Prognostic Utility of Nutritional Risk Index in Patients with Head and Neck Soft Tissue Sarcoma

**DOI:** 10.3390/nu15030641

**Published:** 2023-01-26

**Authors:** Zan Jiao, Chengcai Liang, Guangfeng Luo, Mengmeng Liu, Ke Jiang, Ankui Yang, Yao Liang

**Affiliations:** 1 State Key Laboratory of Oncology in Southern China, Collaborative Innovation Center for Cancer Medicine, Guangzhou 510060, China; 2 Department of Head and Neck Surgery, Sun Yat-sen University Cancer Center, Guangzhou 510060, China; 3 Department of Gastric Surgery, Sun Yat-sen University Cancer Center, Guangzhou 510060, China; 4 Melanoma and Sarcoma Medical Oncology Unit, Sun Yat-sen University Cancer Center, Guangzhou 510060, China

**Keywords:** soft tissue sarcoma, head and neck, nutritional risk index, nomogram, prognosis

## Abstract

Background: The nutritional risk index (NRI) is an excellent indicator of nutritional status and a significant prognostic factor in several malignancies, but the relationship between NRI and the prognosis of head and neck soft tissue sarcoma (HNSTS) patients remains unclear. The aim of this study was to investigate the role of NRI in patients with HNSTS. Methods: We retrospectively reviewed patients with HNSTS between 1990 and 2021. In order to determine the optimal cut-off value of NRI, the Maximally selected log-rank statistic was performed. We evaluated the effect of NRI on overall survival (OS) and progression-free survival (PFS) by using the Kaplan–Meier method and Cox regression analysis. Then, OS and PFS nomograms based on NRI were constructed. Results: In total, 436 HNSTS patients were included in this study. The optimal cut-off value of NRI was 99.34. Patients with low-NRI showed significantly worse OS and PFS than patients with high-NRI, respectively (5-year OS rate of 43.0 vs. 70.8%, 5-year PFS rate of 29.0 vs. 45.0%, all *p* < 0.05). In the multivariate analysis, distant metastasis, deep tumor depth, tumor grade, and NRI were prognostic factors for both PFS and OS, and treatment modality was associated with OS but not PFS. The concordance indexes (C-indexes) of OS and PFS nomograms were 0.794 (95% CI, 0.759–0.829) and 0.663 (95% CI, 0.626–0.700), respectively, which also performed well in the validation set. Conclusions: NRI is an independent predictor of OS and PFS in HNSTS patients. The validated nomograms based on NRI provide useful predictions of OS and PFS for patients with HNSTS.

## 1. Introduction

Head and neck soft tissue sarcoma (HNSTS) arising from mesenchymal cells is a rare and heterogeneous malignancy, accounting for only 1% of head and neck tumors and approximately 10% of soft tissue sarcomas (STS) [1,2,3]. Up to 50 pathological subtypes of HNSTS have been identified, characterized by a conglomerate of anatomical locations and heterogeneous subtypes [4]. HNSTS has more malignancy than STS originating from other body sites that might contribute to poor prognosis of patients [5], with the five-year overall survival (OS) range from 31% to 80% [6]. Traditional prognostic indicators such as histological grade, tumor site, tumor size, and the presence of metastasis have been considered significant predictors of outcome [7,8,9]. However, inaccuracy and inadequacy have been gradually exposed in clinical practice [10]. Therefore, the identification of novel prognostic biomarkers is essential for clinicians to further stratify patients with HNSTS for optimal treatments and improve clinical outcomes.

Accumulating evidence has demonstrated that tumor progression is associated with the status of nutrition [11,12], and malnutrition is common among patients with malignancies [13]. Negative effects of malnutrition have been reported not only in reducing the tolerance and therapeutic efficacy of oncological patients [14] but also in increasing the risk of postoperative complications [15], which could influence the development and outcome of malignancy [16]. Given the highly invasive nature and complicated anatomical location of HNSTS, malnutrition and dysphagia are more likely to occur in patients at the time of diagnosis. Hence, precise assessment of nutritional status is crucial for the prognosis of HNSTS patients. 

Recently, the nutritional risk index (NRI), combining weight, height, and serum albumin levels, has been reported as a simple and sensitive indicator that can objectively evaluate the nutritional status of cancer patients [17]. Owing to its objectivity and comprehensive nature, NRI overcomes the problems of traditional nutritional parameters that consist of inaccuracy and instability, such as body mass index (BMI) and serum albumin (ALB), which could not reflect the long-term nutritional state. Moreover, a large-scale prospective study of nutritional assessment in oral cancer demonstrated that NRI has better accurate prediction compared with BMI or ALB alone [17]. The evaluation of NRI can be performed to predict postoperative outcomes, and assist in planning perioperative nutritional management. However, the relationship between NRI and the prognosis of patients with HNSTS has not been reported.

The aim of the present study was to investigate the role of NRI in patients with HNSTS. Accordingly, we established novel prognostic models based on NRI and significant clinicopathological factors to predict the overall survival (OS) and progression-free survival (PFS) of HNSTS patients.

## 2. Materials and Methods

### 2.1. Study Cohort

We retrospectively reviewed patients who underwent R0 resection for HNSTS at Sun Yat-sen University Cancer Center (SYSUCC) between January 1990 and January 2021 (n = 536). R0 resection was defined as the negative surgical resection margins of macroscopic disease. The inclusion criteria were as follows: (1) confirmed HNSTS by pathological examination; (2) received definitive surgery with R0 resection. The exclusion criteria were as follows: (1) patients who were aged younger than 18 years; (2) missing medical records of height or weight or albumin level; (3) follow-up duration less than 1 year or lost to follow-up; (4) patients receiving any prior treatment; (5) patients with secondary malignancy; (6) patients with any clinical evidence of acute infection or chronic active inflammatory disease or autoimmune disease. After exclusion, a total of 436 patients with confirmed HNSTS and adequate data were included in our study (Supplementary Figure S1). 

This study was approved by the Medical Ethics Committee of SYSUCC. Written informed consent was obtained from all patients in the hospital to offer related information. 

### 2.2. Data Collection

Clinical and pathological information of the patients was collected from the hospital information system, including patient demographics (age, gender, history of smoking exposure, laboratory data, and physical assessment data), pathologic characteristics (tumor site, tumor size, lymph node metastasis, distant metastasis, tumor depth, tumor grade, and TNM stage), treatment modality and follow-up data (follow-up duration, survival, and progression). The laboratory and physical assessment data included serum ALB, height, and weight, which were collected from all subjects in the week preceding surgery. Tumor locations were categorized as scalp or face, neck, nasal cavity or paranasal sinus, oral cavity, pharynx or larynx, and other sites, and were further divided into low-risk sites and high-risk sites depending on the previous studies [5,18]. The TNM stage was classified according to the 7th edition of the American Joint Committee on Cancer (AJCC) [19] and tumor grade was evaluated on the basis of the French Federation of Cancer Centers Sarcoma Group (FNCLCC) systems [7]. All pathological diagnoses of HNSTS were performed by at least two independent experienced pathologists at SYSUCC.

NRI value was calculated by this formula: NRI = 1.487 × serum ALB concentration (g/L) + 41.7 × preoperative weight/ideal body weight (kg) [20]. In this cohort, we defined ideal body weight as 22 × height (m)^2^ [21]. BMI was evaluated as weight (kg)/height (m)^2^ [22]. 

### 2.3. Follow Up

In the first 2 years, all patients were followed up with physical examination and/or magnetic resonance imaging (MRI) every 6 months and then checked annually. Patients with suspicious loco-regional progression on physical examination or MRI underwent a biopsy to confirm pathology. If distant metastasis is suspected, biopsy or serial follow-up imaging should be carried out. 

The primary and secondary endpoints were OS and PFS, respectively. OS was defined as the time from the diagnosis to death from any cause or last follow-up and PFS was defined as the time from the diagnosis to local, regional, or distant progression or death from any cause or latest clinical observation.

### 2.4. Statistical Analysis

Data for continuous variables are expressed as mean (SD) or median and interquartile range (IQR) while data for categorical variables are expressed as absolute numbers and percentages. The Student’s *t*-test was used to compare continuous variables, and the Chi-square test or Fisher’s exact test was used to compare categorical variables. To investigate differences between NRI and baseline characteristics, the optimal cut-off value of NRI was determined by Maximally selected log-rank statistics to categorize the groups. Kaplan–Meier curves with the log-rank test were generated to assess OS and PFS among the whole cohort and different subgroups. Multivariable Cox proportional hazards models were constructed with a univariable inclusion criterion of *p* < 0.05 to identify prognostic factors for mortality and disease progression. Then, we established two nomograms based on the results of multivariate COX regression models with endpoints of 3- and 5-year OS and PFS. The accuracy of predictions was evaluated by estimating the model’s calibration with 1000 bootstrap samples, and discrimination was measured by the concordance index (C-index). Receiver operating characteristic (ROC) curves were used to evaluate the predicting ability of OS and PFS nomograms by measuring the area under the curve (AUC). All statistical tests were conducted using SPSS version 26.0 (SPSS, Chicago, IL, USA) and R version 4.1.2 (http://www.rproject.org, accessed on 10 October 2022). *p* < 0.05 were considered statistically significant for the statistical analyses.

## 3. Results

### 3.1. Baseline Characteristics

As summarized in Table 1, a total of 436 individuals diagnosed with HNSTS were included in this study, including 160 females (36.7%) and 276 males (63.3%), with a mean age of 45.4 ± 15.4 years (range, 18–87 years). The mean BMI was 22.37 ± 3.65 kg/m^2^, with 17.0% having a history of smoking exposure. Tumors were found in the scalp or face (*n* = 129, 29.6%), neck (*n* = 117, 26.8%), nasal cavity or paranasal sinus (*n* = 71, 16.3%), oral cavity (*n* = 49, 11.2%), pharynx or larynx (*n* = 29, 6.7%) and other sites (*n* = 41, 9.4%). The primary tumor size was less than 2.0 cm in 93 patients (21.3%), between 2.0 and 4.0 cm in 153 patients (35.1%), and greater than 4.0 cm in 190 patients (43.6%). Meanwhile, the majority of patients had no lymph node metastases during the initial surgery (*n* = 384, 88.1%), and distant metastases were diagnosed in 24 patients (5.5%). Superficial and deep depth of tumors were found in 157 (36.0%) and 279 (64.0%) patients, respectively. G1 tumors were identified in 173 (39.7%) patients, G2 in 203 (46.5%) patients, and G3 in 60 (13.8%) patients. Most patients (81.4%) had early clinical stage (I/II) according to the TNM stage (AJCC 7th Edition). All patients underwent R0 resection with negative resection margin and 182 patients (41.7%) underwent adjuvant therapy with chemotherapy, radiotherapy, or both. At the end of follow-up, 184 (42.2%) patients had died, and 274 (62.8%) patients had tumor progression since the initial therapy. The median OS and PFS for the entire cohort of 436 patients were 73.6 (IQR: 23.5–73.6) months and 26.2 (IQR: 8.7–26.2) months.

### 3.2. The Association between NRI and Clinical Characteristics

A maximally selected log-rank statistic was applied to establish the optimal NRI cut-off value of prognostic significance. The highest log-rank statistic coincided with a NRI level of 99.34 (Supplementary Figure S2). Based on the cut-off value, the whole cohort was divided into two subgroups: the low-NRI group (NRI ≤ 99.34) and the high-NRI group (NRI > 99.34). The relationship between the NRI and various clinicopathological features were shown in Table 1. There were no differences between the two subgroups in gender, history of smoking, tumor site, tumor size, the status of lymph node metastasis, and treatment modality; however, patients with lower NRI were significantly associated with older age (*p* = 0.003), lower BMI (*p* < 0.001), distant metastasis (*p* = 0.018), deeper depth of tumor (*p* = 0.015), higher tumor grade (*p* = 0.003) and advanced TNM stage (*p* < 0.001). We randomly divided the entire cohort into training and validation sets in the ratio of 7:3, and the baseline characteristics of the training and validation sets are listed in Table 2. The training and validation sets were similar with respect to most variables, and 61 (19.8%) and 29 (22.7%) patients were assigned to the low-NRI groups, respectively.

**Figure 1:**
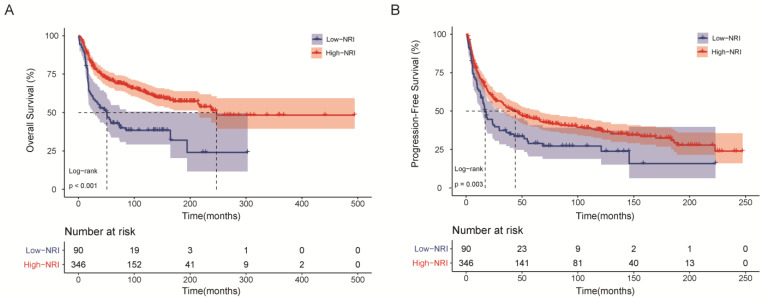
Kaplan–Meier survival curves of HNSTS patients for the whole cohort according to NRI. (**A**) Overall survival; (**B**) Progression-free survival. The log-rank test was used to compare curves. NRI: nutritional risk index.

The Kaplan–Meier survival analysis showed that the low-PNI group was significantly associated with shorter OS relative to the high-NRI group (*p* < 0.001, Figure 1A) and shorter PFS (*p* = 0.003, Figure 1B). The 5-year OS and PFS rates for the high-NRI cohort were 70.8% and 45.0%, while those in the low-NRI cohort were merely 43.0% and 29.0%, respectively. In addition, compared with high-NRI patients, the median (IQR) survival time decreased from 84 (27–149) months to 43 (17–90) months for OS and from 33 (9–96) months to 17 (6–51) months for PFS in the whole cohort.

### 3.3. Factors Predicting OS and PFS Outcomes

We carried out univariate Cox analyses to identify the significant prognostic factors (Supplementary Table S1), and multivariate Cox analyses were performed to assess the independent prognostic factors affecting OS and PFS (Table 3). Age, BMI, tumor site, lymph node metastasis, distant metastasis, tumor depth, tumor grade, TNM stage, treatment modality, and NRI were potential prognostic factors for OS, and these factors without age, BMI, and lymph node metastasis were prognostic factors for PFS (*p* < 0.05 for all). 

In the multivariate analysis, distant metastasis (HR: 5.661, 95% CI: 2.847–11.258, *p* < 0.001), deep tumor depth (HR: 3.306, 95% CI: 1.843–5.929, *p* < 0.001), tumor grade (G2 vs. G1, HR: 4.592, 95% CI: 2.726–7.734, *p* < 0.001; G3 vs. G1, HR: 3.811, 95% CI: 2.034–7.140, *p* < 0.001), treatment modality (Surgery + CT-adjuvant vs. Surgery-definitive, HR: 2.182, 95% CI: 1.269–3.752, *p* = 0.005) and NRI ≤ 99.34 (HR: 1.912, 95% CI: 1.213–3.013, *p* = 0.005) were independent prognostic factors for OS, whereas distant metastasis (HR: 2.609, 95% CI: 1.388–4.907, *p* = 0.003), deep tumor depth (HR: 2.446, 95% CI: 1.663–3.597, *p* < 0.001), tumor grade (G2 vs. G1, HR: 1.613, 95% CI: 1.147–2.267, *p* = 0.006), NRI ≤ 99.3 (HR: 1.581, 95% CI: 1.132–2.210, *p* = 0.007) were independent prognostic factors for PFS.

### 3.4. Nomogram Development and Validation

**Figure 2:**
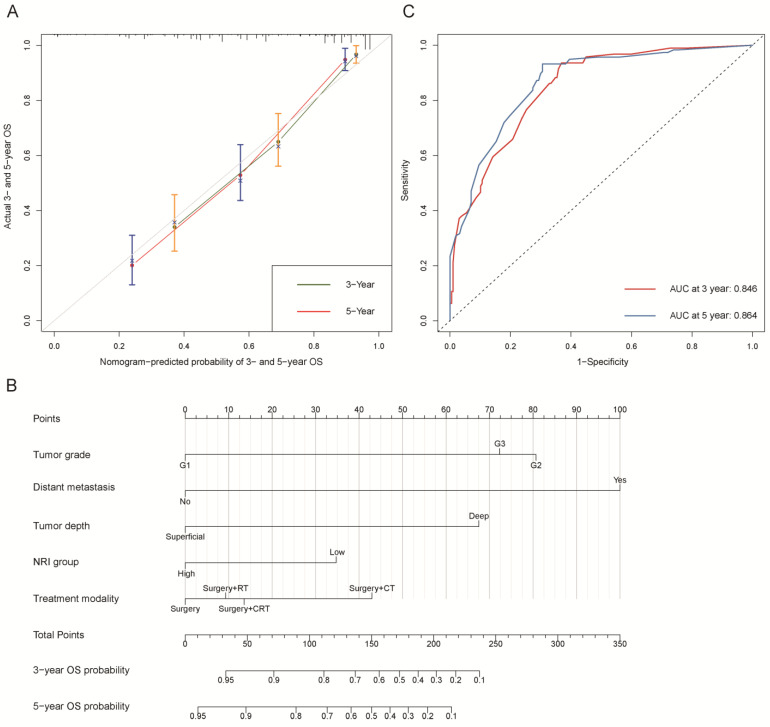
Overall survival (OS). (**A**) Calibration plot of OS at 3- and 5-year. (**B**) Nomogram for predicting the probability of OS at 3- and 5-year. (**C**) Predictive performance of nomogram for OS at 3-year and 5-year, by time-dependent receiver operating characteristic (ROC) curve analysis. The nomogram was constructed by tumor grade, distant metastasis, tumor depth, NRI group, and treatment modality. CT: chemotherapy; RT: radiotherapy; CRT: chemoradiotherapy; NRI group: low group (NRI ≤ 99.34) and high group (NRI > 99.34).

**Figure 3:**
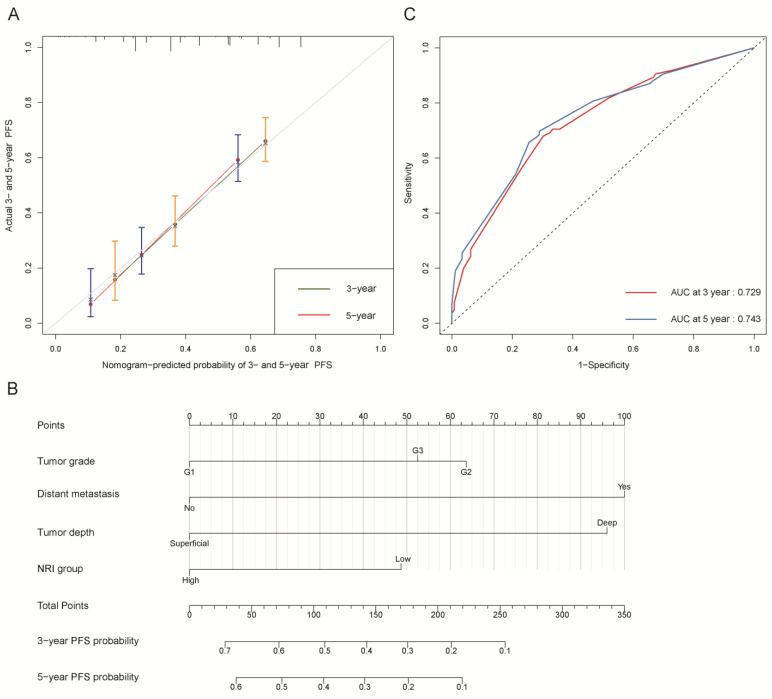
Progression-free survival (PFS). (**A**) Calibration plot of PFS at 3- and 5-year. (**B**) Nomogram for predicting the probability of PFS at 3- and 5-year. (**C**) Predictive performance of nomogram for PFS at 3-year and 5-year, by time-dependent receiver operating characteristic (ROC) curve analysis. The nomogram was constructed by tumor grade, distant metastasis, tumor depth, and NRI group. NRI group: low group (NRI ≤ 99.34) and high group (NRI > 99.34).

We further constructed two nomograms for OS and PFS that integrated independent prognostic factors in the training cohort (Figure 2B and Figure 3B). To estimate 3- and 5-year OS and PFS, the scores for each independent predictor were plotted and serially summed up. 

The C-indexes of OS and PFS nomogram prediction models were 0.794 (95% CI, 0.759–0.829) and 0.663 (95% CI, 0.626–0.700), respectively. The calibration curves (Figure 2A and Figure 3A) adjusted by bootstrapping with 1000 samples for the 3- and 5-year OS and PFS showed a good degree of fit between the predictions and observations in the training cohort. In ROC curves analyses, the AUCs of the nomogram at 3- and 5-year were 0.846 (95% CI, 0.801–0.891) and 0.864 (95% CI, 0.821–0.907) for OS (Figure 2C) and 0.729 (95% CI, 0.672–0.786) and 0.743 (95% CI, 0.684–0.803) for PFS (Figure 3C), respectively.

Good calibration curves were observed for the 3- and 5-year OS and PFS in the validation cohort (Supplementary Figure S3A,B). In the validation cohort, the OS model had a C-index of 0.866 (95% CI, 0.817–0.915) and the PFS model had a C-index of 0.648 (95% CI, 0.583–0.713). As shown in Supplementary Figure S4A,B, the AUCs of the OS model at 3- and 5-year as applied to the validation cohort were 0.864 (95% CI, 0.800–0.929) and 0.893 (95% CI, 0.837–0.950), and 0.713 (95% CI, 0.621–0.804) and 0.713 (95% CI, 0.621–0.805) for PFS model, respectively.

### 3.5. Subgroup Analysis of Common Clinical Variables According to NRI Groups

**Figure 4:**
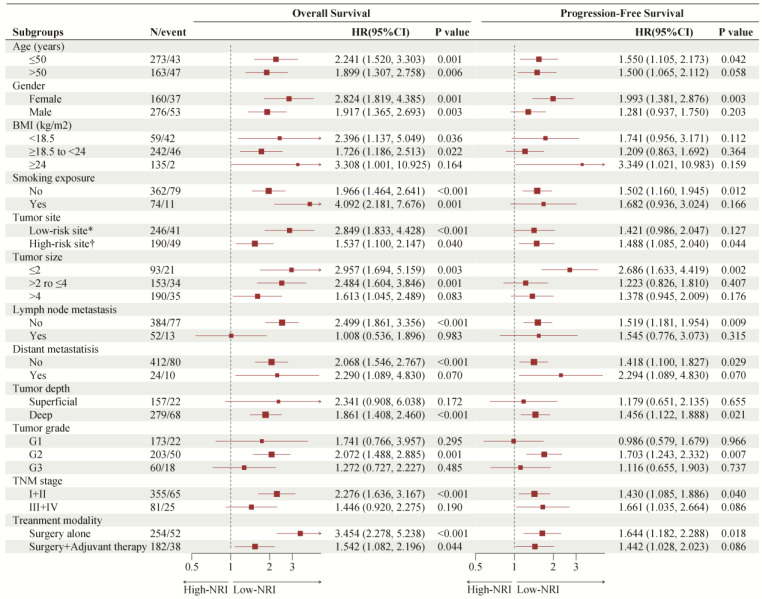
Forest plot of NRI groups on overall survival and progression-free survival in subgroup analyses. NRI: nutritional risk index. * Scalp or face and neck; † Nasal cavity or paranasal sinus, oral cavity, pharynx or larynx, and others.

**Figure 5:**
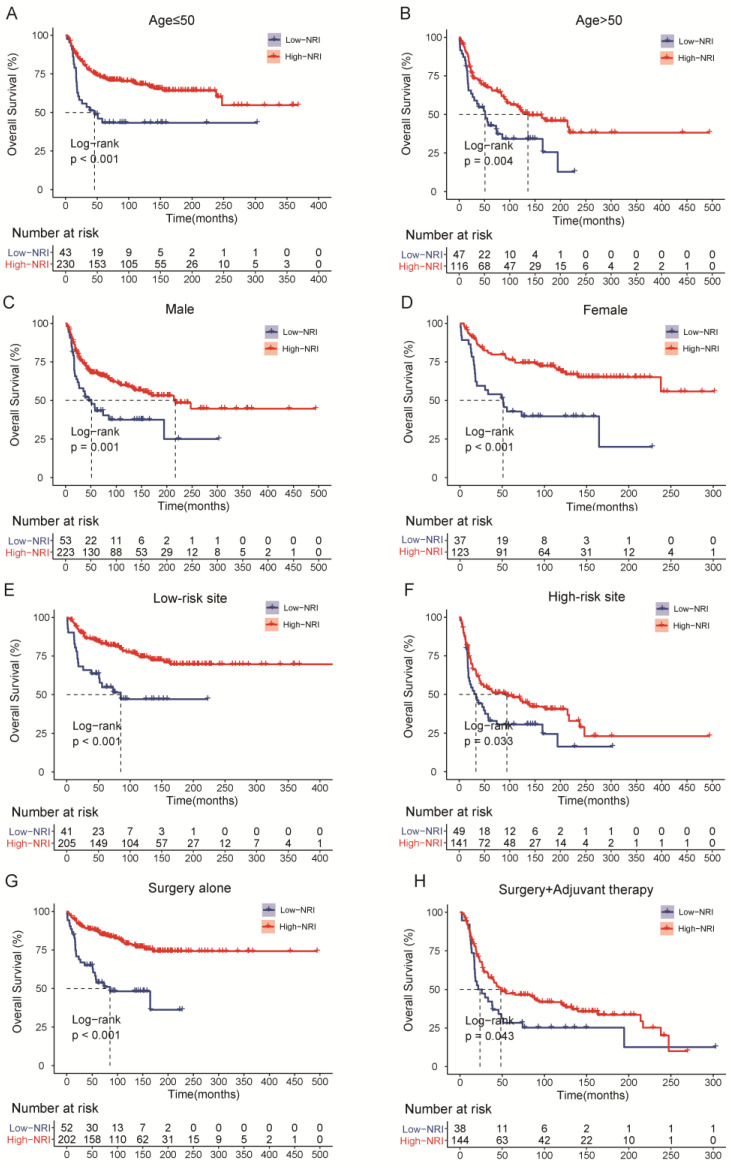
Overall survival (OS) curves of subgroup analysis in HNSTS patients for different types of clinical manifestations according to NRI. (**A**) The OS rate of patients with age ≤ 50; (**B**) The OS rate of patients with age > 50; (**C**) The OS rate of male patients; (**D**) The OS rate of female patients; (**E**) The OS rate of patients with low-risk site; (**F**) The OS rate of patients with high-risk site; (**G**) The OS rate of patients with surgery; (**H**) The OS rate of patients with surgery and adjuvant therapy. NRI: nutritional risk index.

As shown in Figure 4, we investigated the association of PNI with OS and PFS in subgroups of patients with common clinical characteristics. The effect of low-NRI on OS was consistently unfavorable (all hazard ratios, >1.0) across all prespecified subgroups, and the effect of low-NRI on PFS was mostly unfavorable (hazard ratios > 1.0), except for the subgroup of G1. Further Kaplan–Meier curves of clinically meaningful subgroup analysis showed that lower NRI was still significantly related to an increased risk of death in patients with age ≤ 50 or not (Figure 5A,B), in males and females (Figure 5C,D), in patients with low-risk site tumor or high-risk (Figure 5E,F), and in patients with surgery alone or surgery plus adjuvant therapy (Figure 5G,H). Similar results were observed for PFS in Supplementary Figure S5.

## 4. Discussion

To our knowledge, this is the first investigation to identify and validate the prognostic value of NRI in HNSTS patients with curative resection. A low NRI was associated with a shorter OS and PFS in patients with HNSTS. NRI is not only a good indicator of nutritional status in patients but also an independent predictor of mortality and disease progression in HNSTS. Furthermore, the nomograms incorporated NRI were constructed to predict the OS and PFS of patients, which are useful for the personalized management of HNSTS.

Malnutrition is a complex condition, which is characterized by reduced protein reserves, weakened immune defenses, and calorie breakdown [23]. Approximately 20–30% of deaths in patients with cancer were due to malnutrition, rather than cancer itself [24]. Accumulating evidence suggests that malnutrition is related to disease progression and is a major cause of poor therapeutic outcomes [25,26]. The incidence of malnutrition has been reported to be high not only in cancer patients but also in sarcoma patients, and nutritional assessment can effectively reflect the prognosis of patients [27,28,29,30]. Actually, patients with HNSTS are more likely to suffer from the functional restrictions of chewing and swallowing after surgery, because of the special complicated anatomical location of HNSTS and postoperative complications. Additionally, nausea and vomiting symptoms are more often seen in head and neck malignancies, especially for the patients who received adjuvant therapy, which would exacerbate any malnutrition [31]. In the subgroup analysis of patients with surgery and adjuvant therapy, low-NRI continued to be predictive of shorter OS, demonstrating its good clinical applicability. Overall, malnutrition is more prevalent among HNSTS patients. Hence, NRI, as an excellent indicator of the nutritional status of surgical patients [32], is becoming more and more crucial for improving the quality of HNSTS patients, and it deserves sufficient consideration. Of note, it provides a long-term trend of the nutritional status of patients by integrating body weight, height, and serum ALB, which can remain stable in the short term [33]. Interestingly, a previous study based on weight loss, BMI, and NRI was reported by Righini et al., revealing that approximately 49% of patients with HNSC were at the status of malnutrition, and patients with oral or oropharyngeal tumor sites were more likely to suffer from malnutrition [34]. However, among the tumor sites of HNSTS, our study dividing the nutritional status based on NRI showed that the scalp or face and neck sites were the most common sites. The malnourished proportion of oral and oropharyngeal HNSTS was only 26.5% and 34.5%, and there was no statistical significance with NRI according to tumor sites, probably because HNSTS has the heterogeneous malignancy with different subtypes and distinct biological behaviors.

In the present study, we found that a low NRI level was significantly associated with older age, lower BMI, distant metastases, deeper depth of tumor, higher tumor grade, and advanced TNM stage, indicating that there is a potential correlation between aggressive and advanced tumors and the reduction of nutrient levels. Similar to our finding, several reports also found that patients with malnutrition are at a higher probability of aggressive head and neck cancer [35,36]. In addition, Nakayama et al. showed that patients with low NRI levels were at higher risk of advanced clinical stage and mortality [37]. The reason for the relationship between low NRI levels and advanced tumors might be due to the various cytokines released by the advanced tumor [38,39,40]. Moreover, several studies have shown that elevated inflammatory factors and poor nutrient absorption are associated with increased catabolism, leading to anorexia and malnutrition [41,42,43]. Older patients with lower BMI are more likely to have a low level of NRI, highlighting the importance of attention to the nutritional status of older patients.

This study also showed that distant metastasis, deep tumor depth, tumor grade, and NRI were prognostic factors for both PFS and OS, and treatment modality was associated with OS but not PFS. Of note, after multivariate adjustment for prognostic factors, NRI remained the independent prognostic factor both for OS and PFS. Similarly, Jung et al. conducted related research and found that age, tumor grade, tumor size, and initial distant metastasis were independent predictors for OS, and tumor size and tumor grade were independent predictors for PFS [44]. Moreover, a large-scale prospective study for the nutritional assessment of oral cancer demonstrated that patients with low levels of NRI were associated with worse prognosis in oral cancer and the prognostic performance of NRI was superior to BMI or serum ALB [17]. Nevertheless, there are minor differences with our study, probably because of the genomic heterogeneity between these malignancies, but our study with longer follow-up time and larger sample size allowed us to better identify the risk assessment of NRI and conventional factors for OS and PFS outcomes. 

More importantly, this is the first study to demonstrate the correlation of NRI level with OS and PFS in HNSTS. The Kaplan–Meier survival analysis showed that the 5-year OS rate of patients with low-NRI was nearly 30% lower than those of patients with high-NRI (43.0% vs. 70.8%) and the 5-year PFS rate of patients with low-NRI was nearly 16% lower than those of patients with high-NRI (29.0% vs. 45.0%), suggesting that patients who have low levels of NRI should require greater scrutiny and closer follow-up after initial treatment. However, the mechanism of NRI influencing the prognosis of patients with HNSTS is not clear.

In our subgroup analysis, evidence of the higher risk of mortality and disease progression in patients with low-NRI, as compared with the high-NRI group, was that the effects of low-NRI were consistently unfavorable with each subgroup. Based on these findings, further Kaplan–Meier curves also demonstrated that patients with low-NRI, grouped by age, gender, tumor site, and treatment modality, tended to have shorter OS and PFS, which increase the clinical applicability of NRI. Considering that this was a subgroup analysis, the results should be interpreted with caution and confirmed with further large sample studies.

Based on NRI and other independent risk factors, we established and validated novel prognostic nomograms to predict the 3- and 5-year OS and PFS for HNSTS patients treated with primary therapy. Notably, OS and PFS nomograms showed good discrimination and calibration in both training and validation cohorts, indicating excellent discriminative ability and general applicability. As compared with the previously published nomograms for HNSTS [45], which had the C-indexes of 0.748 for OS and 0.644 for PFS, our nomograms have superior effectiveness and predictive accuracy. Most importantly, our nomograms integrating significant clinicopathologic characteristics, treatment modality and nutritional status can be used to predict personalized estimates of OS and PFS, which can help stratify HNSTS patients precisely and provide discussions of prognosis.

As the NRI is a valid tool for the assessment of the nutritional status of patients, our study demonstrated that NRI is an independent prognostic factor in patients with HNSTS, which is consistent with several previous studies in liver cancer [46], breast cancer [47], gastric cancer [48] and esophageal squamous cell cancer [49]. While the conclusions of these studies were similar with respect to NRI, they differed slightly in the cut-off value. The possible reasons for the difference in cut-off value are the unique biological characteristics of different malignant tumors and different statistical methods for calculating the optimal cut-off point value. The findings of this study suggest that the comprehensive nutritional assessment with NRI may be essential for the individualized management of HNSTS patients. Therefore, in addition to the routine treatment, we should pay more attention to the levels of NRI for HNSTS patients.

There are some limitations in this study. First, due to the rarity of HNSTS, the study was conducted retrospectively with a limited number of patients from a single center, making it susceptible to the inherent biases of such a study format, although our study represents one of the largest cohorts of patients with HNSTS. Missing data and loss to follow-up may have introduced inherent biases, which is a limitation of our study. Therefore, future prospective multicenter studies are required to corroborate our findings. Second, numerous predictive biomarkers have been validated in many malignancies, such as the controlling nutritional status (CONUT) score [50], Glasgow prognostic score (GPS) [51], and prognostic nutritional index (PNI) [52], which could be further evaluated in the future study. Third, because of the histopathologic diversity, especially for the diverse heterogeneous subtypes of HNSTS, our cohort did not allow us to stratify the analysis of histopathologic subtypes. Nevertheless, our study enhances the clinical utility of NRI in rare malignancies.

## 5. Conclusions

In conclusion, NRI is an independent predictor of OS and PFS in HNSTS patients, which could be utilized for predicting the prognosis of patients with HNSTS in daily clinical practice. Moreover, the validated nomograms provide useful predictions of OS and PFS for patients with HNSTS, guiding clinicians to assess prognosis and develop a personalized treatment strategy. 

## Figures and Tables

**Table 1:** Characteristics of patients classified according to NRI.

Characteristics	Total, No. (%)	Low-NRI Group, No. (%)	High-NRI Group, No. (%)	*p*-Value
	(*n* = 436)	(*n* = 90)	(*n* = 346)	
Age (years, mean ± SD)	45.4 ± 15.4	49.6 ± 17.1	44.3 ± 14.7	0.003
Gender				0.394
Female	160 (36.7)	37 (41.1)	123 (35.6)	
Male	276 (63.3)	53 (58.9)	223 (64.4)	
BMI (kg/m^2^, mean ± SD)	22.37 ± 3.65	19.00 ± 2.31	23.24 ± 3.41	<0.001
Smoking exposure				0.234
No	362 (83.0)	79 (87.8)	283 (81.8)	
Yes	74 (17.0)	11 (12.2)	63 (18.2)	
Tumor site				0.155
Scalp or face	129 (29.6)	20 (22.2)	109 (31.5)	
Neck	117 (26.8)	21 (23.3)	96 (27.7)	
Nasal cavity or paranasal sinus	71 (16.3)	18 (20.0)	53 (15.3)	
Oral cavity	49 (11.2)	13 (14.4)	36 (10.4)	
Pharynx or larynx	29 (6.7)	10 (11.1)	19 (5.5)	
Others	41 (9.4)	8 (8.9)	33 (9.5)	
Tumor size (cm)				
≤2	93 (21.3)	21 (23.3)	72 (20.8)	0.601
>2 to ≤4	153 (35.1)	34 (37.8)	119 (34.4)	
>4	190 (43.6)	35 (38.9)	155 (44.8)	
Lymph node metastasis				
No	384 (88.1)	77 (85.6)	307 (88.7)	0.519
Yes	52 (11.9)	13 (14.4)	39 (11.3)	
Distant metastasis				
No	412 (94.5)	80 (88.9)	332 (95.9)	0.018
Yes	24 (5.5)	10 (11.1)	14 (4.1)	
Tumor depth				0.015
Superficial	157 (36.0)	22 (24.4)	135 (39.0)	
Deep	279 (64.0)	68 (75.6)	211 (61.0)	
Tumor grade				
G1	173 (39.7)	22 (24.4)	151 (43.7)	0.003
G2	203 (46.5)	50 (55.6)	153 (44.2)	
G3	60 (13.8)	18 (20.0)	42 (12.1)	
TNM stage (AJCC7)				
I	150 (34.4)	16 (17.8)	134 (38.7)	<0.001
II	205 (47.0)	49 (54.4)	156 (45.1)	
III	58 (13.3)	15 (16.7)	43 (12.4)	
IV	23 (5.3)	10 (11.1)	13 (3.8)	
Treatment modality				0.816
Surgery-definitive	254 (58.3)	52 (57.8)	202 (58.4)	
Surgery + CT-adjuvant	38 (8.7)	6 (6.7)	32 (9.2)	
Surgery + RT-adjuvant	72 (16.5)	17 (18.9)	55 (15.9)	
Surgery + CRT-adjuvant	72 (16.5)	15 (16.6)	57 (16.5)	

Abbreviations: NRI: nutritional risk index; SD: standard deviation; BMI: body mass index; AJCC7: American Joint Committee on Cancer, 7th Edition; CT: chemotherapy; RT: radiotherapy; CRT: chemoradiotherapy.

**Table 2:** The baseline characteristics in the Training and Validation sets.

Characteristics	Training Set, No. (%)	Validation Set, No. (%)	*p*-Value
	(n = 308)	(n = 128)	
Age (years, mean ± SD)	45.0 ± 15.0	46.2 ± 16.3	0.467
Gender			0.748
Female	115 (37.3)	45 (35.2)	
Male	193 (62.7)	83 (64.8)	
BMI (kg/m^2^, mean ± SD)	22.44 ± 3.71	22.18 ± 3.50	0.501
Smoking exposure			1.000
No	256 (83.1)	106 (82.8)	
Yes	52 (16.9)	22 (17.2)	
Tumor site			0.135
Scalp or face	95 (30.8)	34 (26.6)	
Neck	73 (23.7)	44 (34.4)	
Nasal cavity or paranasal sinus	55 (17.8)	16 (12.5)	
Oral cavity	33 (10.7)	16 (12.5)	
Pharynx or larynx	24(7.8)	5 (3.9)	
Others	28 (9.1)	13 (10.1)	
Tumor size (cm)			0.814
≤2	68 (22.1)	25 (19.5)	
>2 to ≤4	106 (34.4)	47 (36.7)	
>4	134 (43.5)	56 (43.8)	
Lymph node metastasis			0.294
No	275 (89.3)	109 (85.2)	
Yes	33 (10.7)	19 (14.8)	
Distant metastasis			0.476
No	289 (93.8)	123 (96.1)	
Yes	19 (6.2)	5 (3.9)	
Tumor depth			0.758
Superficial	109 (35.4)	48 (37.5)	
Deep	199 (64.6)	80 (62.5)	
Tumor grade			
G1	116 (37.7)	57 (44.5)	0.164
G2	144 (46.7)	59 (46.1)	
G3	48 (15.6)	12 (9.4)	
TNM stage (AJCC7)			
I	100 (32.5)	50 (39.1)	0.297
II	150 (48.7)	55 (43.0)	
III	39 (12.6)	19 (14.8)	
IV	19 (6.2)	4 (3.1)	
Treatment modality			0.044
Surgery-definitive	167 (54.2)	87 (68.0)	
Surgery + CT-adjuvant	32 (10.4)	6 (4.7)	
Surgery + RT-adjuvant	54 (17.85	18 (14.1)	
Surgery + CRT-adjuvant	55 (17.9)	17 (13.3)	
NRI			0.779
≤99.34	62 (20.1)	28 (21.9)	
>99.34	246 (79.9)	100 (78.1)	

Abbreviations: SD: standard deviation; BMI: body mass index; AJCC7: American Joint Committee on Cancer, 7th Edition; CT: chemotherapy; RT: radiotherapy; CRT: chemoradiotherapy; NRI: nutritional risk index.

**Table 3:** Multivariate Cox regression analysis of factors associated with overall survival and progression-free survival.

Characteristics	Overall Survival		Progression-Free Survival	
	HR (95% CI)	*p*-Value	HR (95% CI)	*p*-Value
Age (years)	1.006 (0.994–1.019)	0.325	-	-
BMI (kg/m^2^)	1.000 (0.943–1.060)	0.997	-	-
Tumor site				
Low-risk site *	1.0 [Reference]		1.0 [Reference]	
High-risk site †	1.057 (0.726–1.538)	0.774	0.756 (0.547–1.046)	0.091
Lymph node metastasis				
No	1.0 [Reference]		-	-
Yes	1.177 (0.602–2.302)	0.633	-	-
Distant metastasis				
No	1.0 [Reference]		1.0 [Reference]	
Yes	5.661 (2.847–11.258)	<0.001	2.609 (1.388–4.907)	0.003
Tumor depth				
Superficial	1.0 [Reference]		1.0 [Reference]	
Deep	3.306 (1.843–5.929)	<0.001	2.446 (1.663–3.597)	<0.001
Tumor grade				
G1	1.0 [Reference]		1.0 [Reference]	
G2	4.592 (2.726–7.734)	<0.001	1.613 (1.147–2.267)	0.006
G3	3.811 (2.034–7.140)	<0.001	1.544 (0.972–2.455)	0.066
TNM stage (AJCC7)				
I + II	1.0 [Reference]		1.0 [Reference]	
III + IV	1.081 (0.563–2.077)	0.814	0.792 (0.496–1.265)	0.330
Treatment modality				
Surgery-definitive	1.0 [Reference]		1.0 [Reference]	
Surgery + CT-adjuvant	2.182 (1.269–3.752)	0.005	1.470 (0.893–2.417)	0.130
Surgery + RT-adjuvant	1.164 (0.729–1.859)	0.525	1.162 (0.788–1.712)	0.449
Surgery + CRT-adjuvant	1.237 (0.741–2.066)	0.416	1.281 (0.836–1.963)	0.256
NRI				
>99.34	1.0 [Reference]		1.0 [Reference]	
≤99.34	1.912 (1.213–3.013)	0.005	1.581 (1.132–2.210)	0.007

Abbreviations: BMI: body mass index; AJCC7: American Joint Committee on Cancer, 7th Edition; CT: chemotherapy; RT: radiotherapy; CRT: chemoradiotherapy; NRI: nutritional risk index; * Scalp or face and neck. † Nasal cavity or paranasal sinus, oral cavity, pharynx or larynx, and others.

## Data Availability

The data presented in this study are available on request from the corresponding author.

## Supplementary Materials

The following supporting information can be downloaded at: https://www.mdpi.com/article/10.3390/nu15030641/s1, Figure S1: Flowchart of the patient selection process. HNSTS, head, and neck soft tissue sarcoma; SYSUCC, Sun Yat-sen University Cancer Center; Figure S2: The maximum of the standardized log-rank statistics for NRI cut-off value; Figure S3: Calibration plot of OS (A) and PFS (B) at 3- and 5-year in the validation cohort; Figure S4: Predictive performance of nomogram for OS (A) and PFS (B) at 3- and 5-year, by time-dependent receiver operating characteristic (ROC) curve analysis; Figure S5: Progression-free survival (PFS) curves of subgroup analysis in HNSTS patients for different types of clinical manifestations according to NRI. (A) The PFS rate of patients with age ≤ 50; (B) The PFS rate of patients with age > 50; (C) The PFS rate of male patients; (D) The PFS rate of female patients; (E) The PFS rate of patients with low-risk site; (F) The PFS rate of patients with high-risk site; (G) The PFS rate of patients with surgery; (H) The PFS rate of patients with surgery and adjuvant therapy. NRI: nutritional risk index. Table S1: Univariate Cox regression analysis of factors associated with overall survival and progression-free survival.

## Abbreviations

HNSTS	head and neck soft tissue sarcoma
STS	soft tissue sarcoma
NRI	nutritional risk index
BMI	body mass index
ALB	serum albumin
OS	overall survival
PFS	progression-free survival
SYSUCC	Sun Yat-sen University Cancer Center
AJCC	American Joint Committee on Cancer
FNCLCC	French Federation of Cancer Centers Sarcoma Group
MRI	magnetic resonance imaging
IQR	interquartile range
SD	standard deviation
C-index	concordance index
AUC	area under the curve
ROC	receiver operating characteristic
CONUT	controlling nutritional status
GPS	Glasgow prognostic score
PNI	prognostic nutritional index
