# Supplementary material for: Prognostic Utility of Nutritional Risk Index in Patients with Head and Neck Soft Tissue Sarcoma
Source: Nutrients. 2023 Jan 26;15(3):641. doi: 10.3390/nu15030641 (PMC9920856; doi:10.3390/nu15030641)
Supplement: Supplementary file 1 [file nutrients-15-00641-s001.zip › Table S1.docx]

| Table S1. Univariate Cox regression analysis of factors associated with overall survival and progression-free survival | | | | | |
| --- | --- | --- | --- | --- | --- |
| **Characteristics** | **Overall Survival** | |  | **Progression-Free Survival** | |
|  | HR (95% CI) | ***p*-value** |  | **HR (95% CI)** | ***p*-value** |
| **Age (years)** | 1.015 (1.004-1.027) | 0.007 |  | 1.002 (0.993-1.011) | 0.691 |
| **Gender** |  |  |  |  |  |
| Female | 1.0 [Reference] |  |  | 1.0 [Reference] |  |
| Male | 1.344 (0.943-1.916) | 0.102 |  | 1.209 (0.907-1.613) | 0.196 |
| BMI (kg/m^2^) | 0.951 (0.906-0.998) | 0.040 |  | 0.979 (0.941-1.018) | 0.284 |
| Smoking exposure |  |  |  |  |  |
| No | 1.0 [Reference] |  |  | 1.0 [Reference] |  |
| Yes | 1.243 (0.830-1.862) | 0.291 |  | 1.323 (0.939-1.865) | 0.110 |
| Tumor site |  |  |  |  |  |
| Low-risk site* | 1.0 [Reference] |  |  | 1.0 [Reference] |  |
| High-risk site† | 2.134 (1.521-2.992) | <0.001 |  | 1.475 (1.119-1.945) | 0.006 |
| Tumor size (cm) |  |  |  |  |  |
| ≤2 | 1.0 [Reference] |  |  | 1.0 [Reference] |  |
| >2 to ≤4 | 0.899 (0.574-1.407) | 0.641 |  | 1.178 (0.804-1.726) | 0.399 |
| >4 | 1.014 (0.663-1.549) | 0.950 |  | 1.218 (0.844-1.757) | 0.292 |
| Lymph node metastasis |  |  |  |  |  |
| No | 1.0 [Reference] |  |  | 1.0 [Reference] |  |
| Yes | 2.313 (1.497-3.575) | <0.001 |  | 1.070 (0.673-1.700) | 0.776 |
| Distant metastasis |  |  |  |  |  |
| No | 1.0 [Reference] |  |  | 1.0 [Reference] |  |
| Yes | 6.654 (4.038-10.963) | <0.001 |  | 2.606 (1.616-4.200) | <0.001 |
| Tumor depth |  |  |  |  |  |
| Superficial | 1.0 [Reference] |  |  | 1.0 [Reference] |  |
| Deep | 6.096 (3.566-10.422) | <0.001 |  | 2.691 (1.934-3.746) | <0.001 |
| Tumor grade |  |  |  |  |  |
| G1 | 1.0 [Reference] |  |  | 1.0 [Reference] |  |
| G2 | 5.294 (3.247-8.632) | <0.001 |  | 1.920 (1.399-2.634) | <0.001 |
| G3 | 6.096 (3.487-10.658) | <0.001 |  | 2.154 (1.435-3.234) | <0.001 |
| TNM stage (AJCC7) |  |  |  |  |  |
| I+II | 1.0 [Reference] |  |  | 1.0 [Reference] |  |
| III+IV | 3.033 (2.122-4.336) | <0.001 |  | 1.563 (1.114-2.193) | 0.010 |
| Treatment modality |  |  |  |  |  |
| Surgery-definitive | 1.0 [Reference] |  |  | 1.0 [Reference] |  |
| Surgery + CT-adjuvant | 4.327 (2.595-7.214) | <0.001 |  | 2.046 (1.284-3.262) | 0.003 |
| Surgery + RT-adjuvant | 2.403 (1.548-3.733) | <0.001 |  | 1.746 (1.220-2.498) | 0.002 |
| Surgery + CRT-adjuvant | 3.345 (2.173-5.148) | <0.001 |  | 1.925 (1.336-2.775) | <0.001 |
| NRI |  |  |  |  |  |
| >99.34 | 1.0 [Reference] |  |  | 1.0 [Reference] |  |
| ≤99.34 | 2.263 (1.574-3.253) | <0.001 |  | 1.769 (1.282-2.442) | 0.001 |
| Abbreviations: BMI: body mass index; AJCC7: American Joint Committee on Cancer, 7th Edition; CT: chemotherapy; RT: radiotherapy; CRT: chemoradiotherapy; NRI: nutritional risk index. *Scalp or face and neck. †Nasal cavity or paranasal sinus, oral cavity , pharynx or larynx and others. | | | | | |
