# Supplementary figures and images for: Prognostic Utility of Nutritional Risk Index in Patients with Head and Neck Soft Tissue Sarcoma
Source: Nutrients. 2023 Jan 26;15(3):641. doi: 10.3390/nu15030641 (PMC9920856; doi:10.3390/nu15030641)

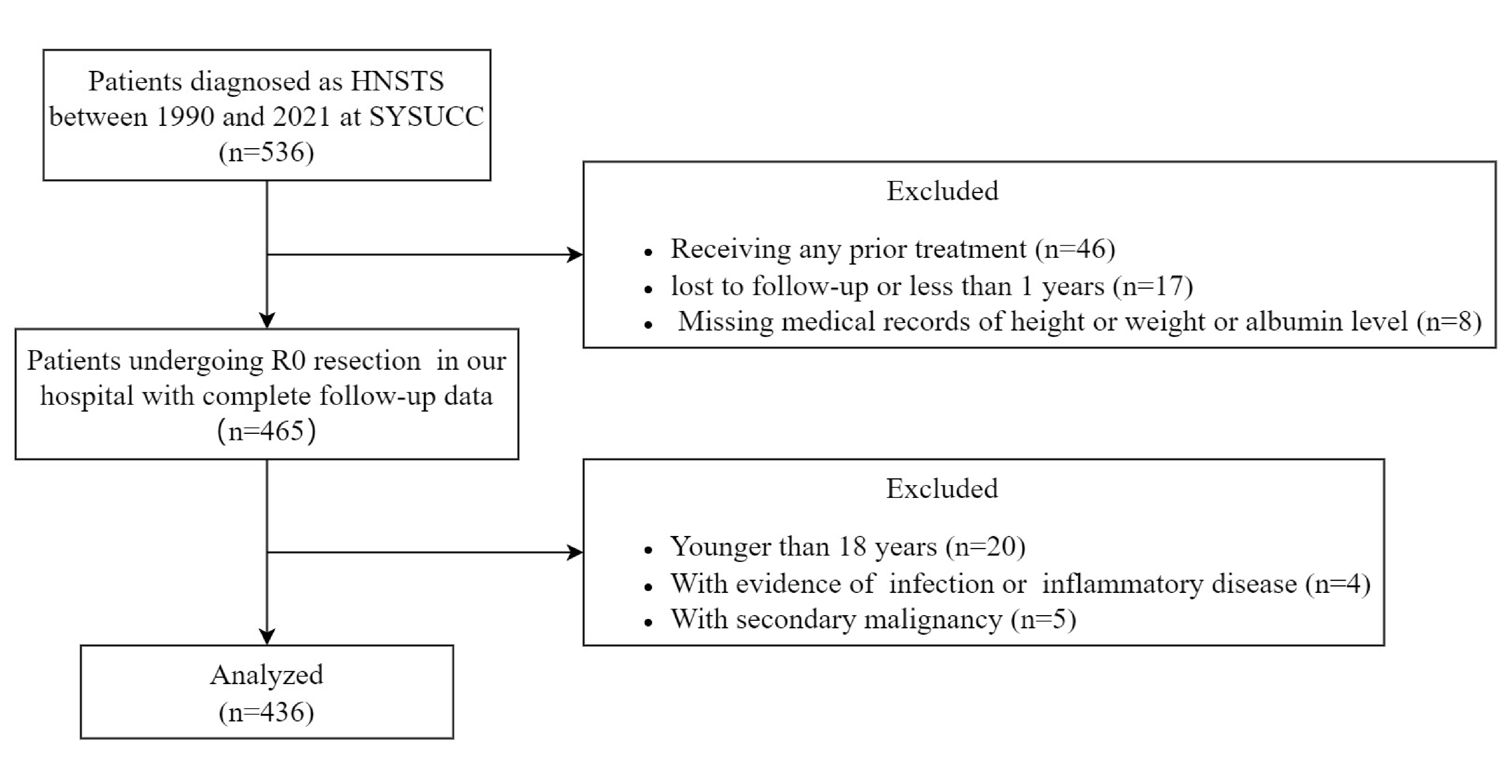

Supplement: Supplementary file 1 [file nutrients-15-00641-s001.zip › Figure S1.tif]

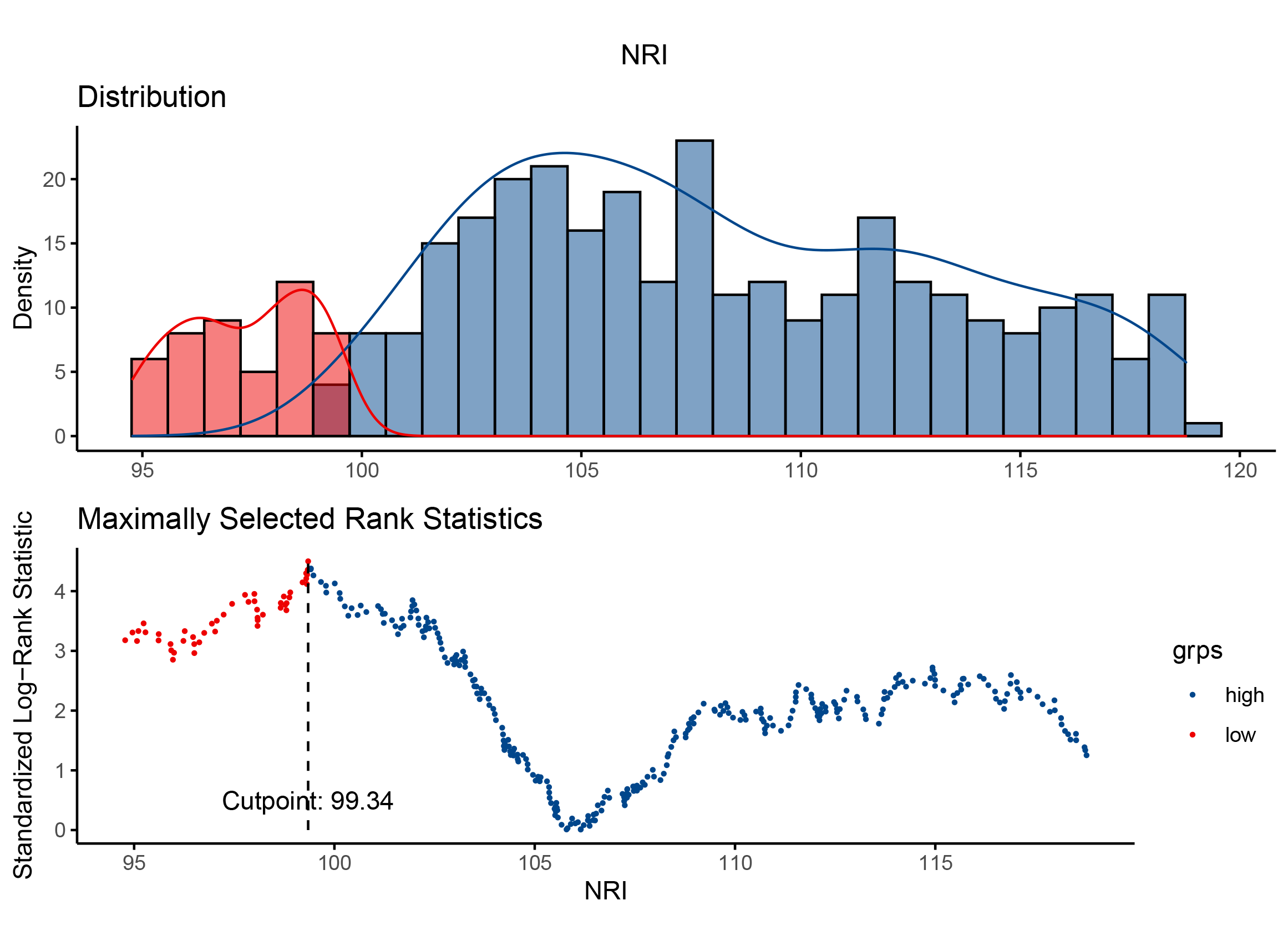

Supplement: Supplementary file 1 [file nutrients-15-00641-s001.zip › Figure S2.tif]

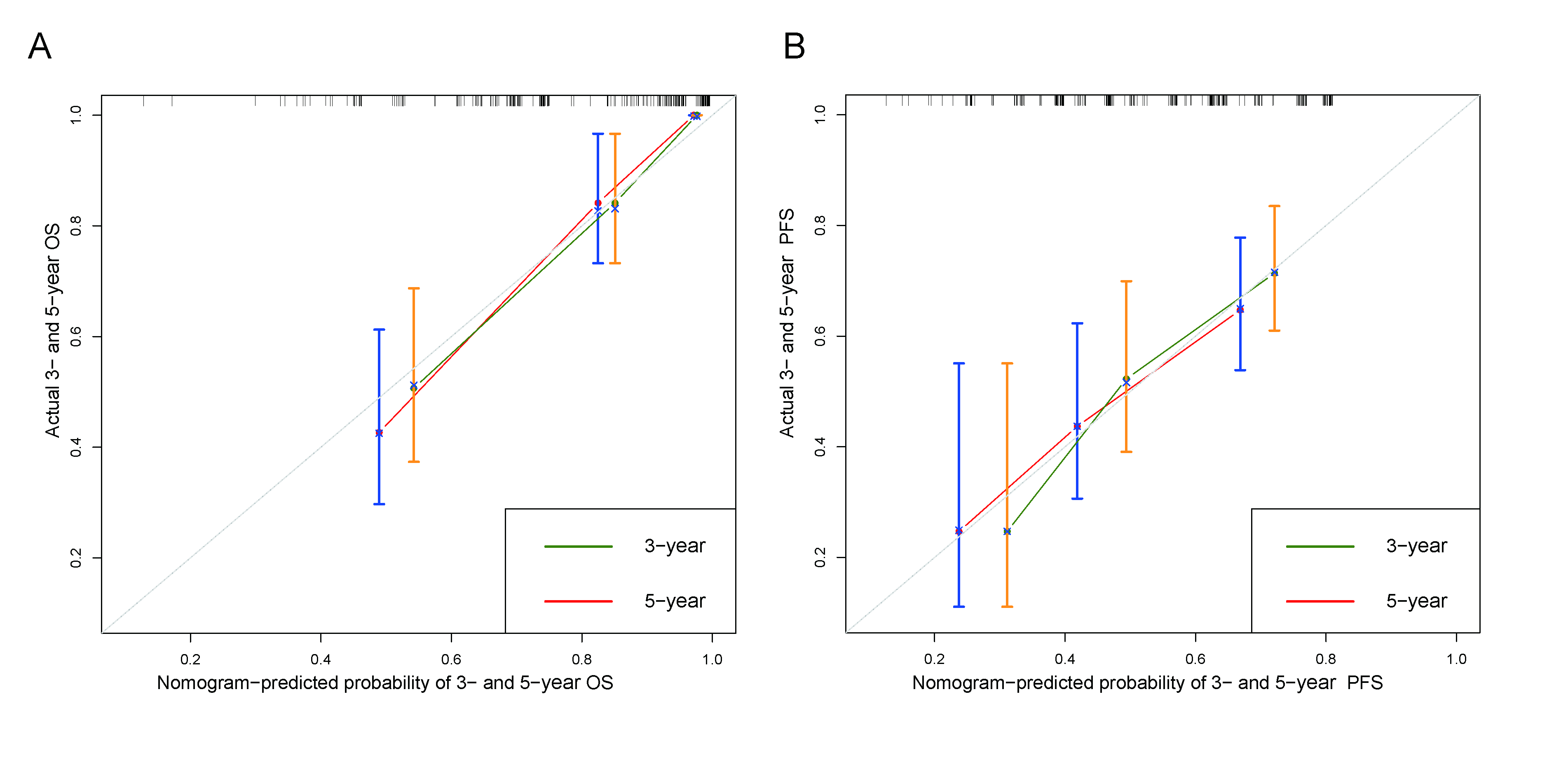

Supplement: Supplementary file 1 [file nutrients-15-00641-s001.zip › Figure S3.tif]

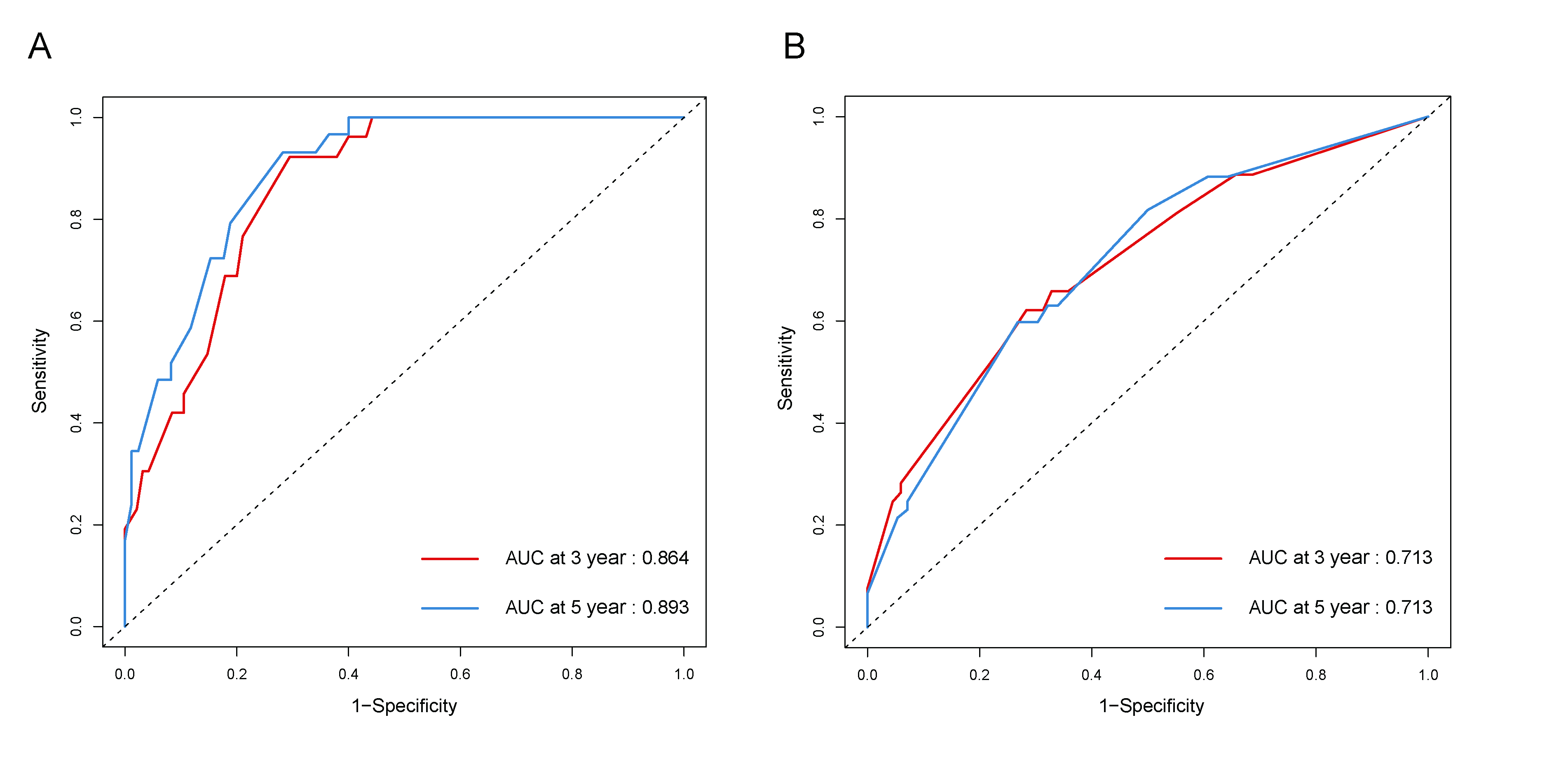

Supplement: Supplementary file 1 [file nutrients-15-00641-s001.zip › Figure S4.tif]

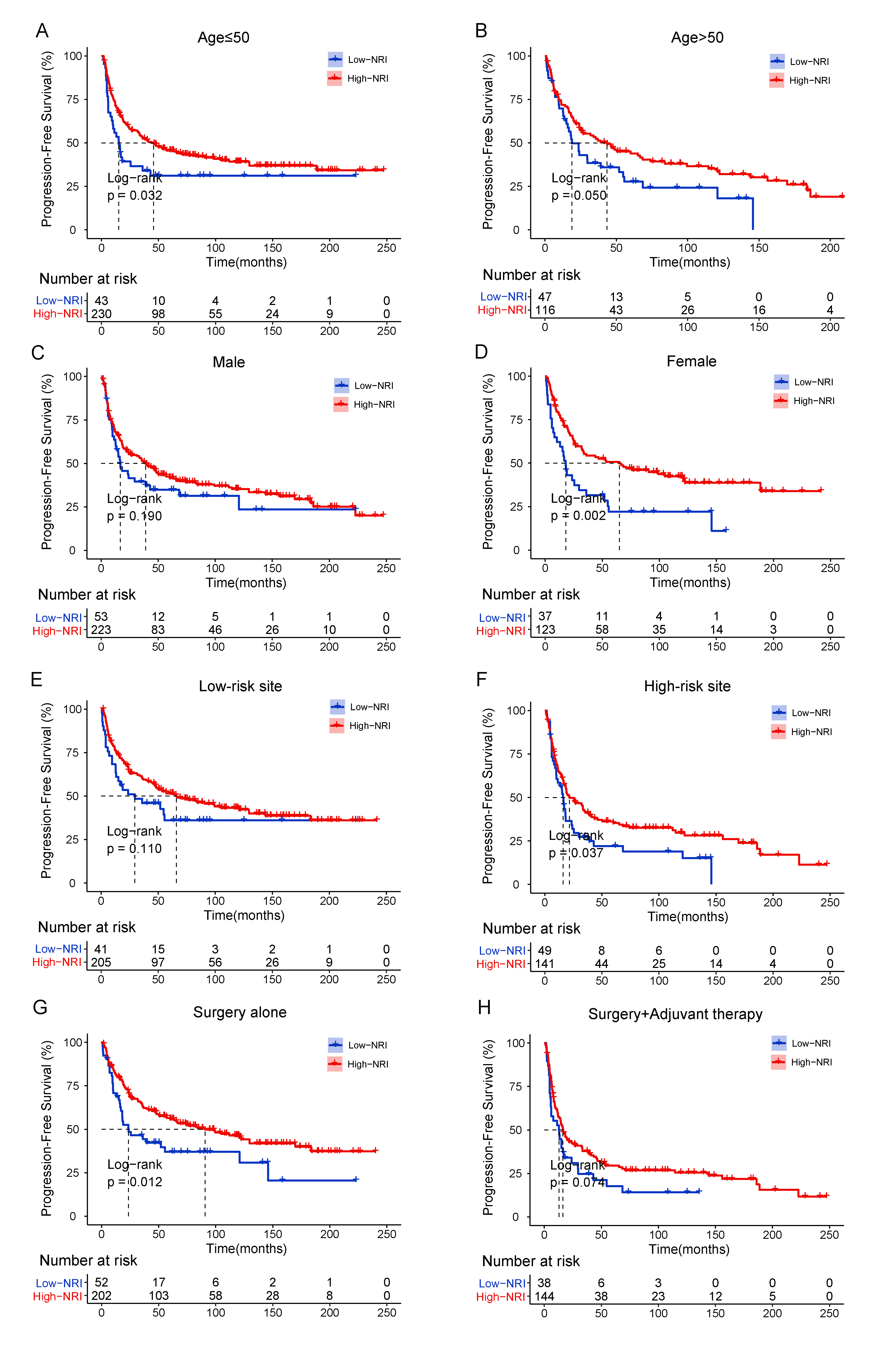

Supplement: Supplementary file 1 [file nutrients-15-00641-s001.zip › Figure S5.tif]
